# The trifunctional antibody catumaxomab for the treatment of malignant ascites due to epithelial cancer: Results of a prospective randomized phase II/III trial

**DOI:** 10.1002/ijc.25423

**Published:** 2010-04-27

**Authors:** Markus M Heiss, Pawel Murawa, Piotr Koralewski, Elzbieta Kutarska, Olena O Kolesnik, Vladimir V Ivanchenko, Alexander S Dudnichenko, Birute Aleknaviciene, Arturas Razbadauskas, Martin Gore, Elena Ganea-Motan, Tudor Ciuleanu, Pauline Wimberger, Alexander Schmittel, Barbara Schmalfeldt, Alexander Burges, Carsten Bokemeyer, Horst Lindhofer, Angelika Lahr, Simon L Parsons

**Affiliations:** 1 Department of Surgery, Cologne-Merheim Medical Center, University of Witten-HerdeckeCologne, Germany; 2 Department of Oncological Surgery, Wielkoposka Cancer CenterPoznan, Poland; 3 Department of Chemotherapy, Rydygier Memorial HospitalKrakow, Poland; 4 Department of Oncological Gynecology, Center of Oncology of LublinLublin, Poland; 5 Institute of Oncology, Academy of Medical Science of UkraineKiev, Ukraine; 6 Regional Clinical Oncology Dispensary, Velikiy NovgorodRussia; 7 Department of Oncology and Pediatric Oncology, Kharkov Medical Academy of Postgraduate EducationKharkov, Ukraine; 8 Institute of Oncology, Vilnius UniversityVilnius, Lithuania; 9 Department of Surgery, Klaipeda Seamen's HospitalKlaipeda, Lithuania; 10 Medical Oncology and Drug Development, Royal Marsden HospitalLondon, United Kingdom; 11 Department of Oncology, Spitalul Judetean de Urgenta ‘Sf Ioan cel Nou’Suceava, Romania; 12 Department of Medical Oncology, Cancer Institute Ion ChiricutaCluj-Napoca, Romania; 13 Department for Obstetrics and Gynecology, University of Duisburg-EssenEssen, Germany; 14 Department of Hematology, Oncology and Transfusion Medicine, Charité, University Hospital BerlinBerlin, Germany; 15 Department for Obstetrics and Gynecology, Technical University MunichMunich, Germany; 16 Department of Obstetrics and Gynecology, University Hospital GrosshadernMunich, Germany; 17 Department of Internal Medicine II (Hematology, Oncology), University Hospital EppendorfHamburg, Germany; 18 TRION Pharma GmbHMunich, Germany; 19 Fresenius Biotech GmbHMunich, Germany; 20 Department of Surgery, Nottingham University Hospitals NHS TrustNottingham, United Kingdom

**Keywords:** catumaxomab, trifunctional antibody, malignant ascites, epithelial cancer, clinical trial

## Abstract

Malignant ascites is a common manifestation of advanced cancers, and treatment options are limited. The trifunctional antibody catumaxomab (anti-epithelial cell-adhesion molecule x anti-CD3) represents a targeted immunotherapy for the intraperitoneal (i.p.) treatment of malignant ascites secondary to epithelial cancers. In this phase II/III trial (EudraCT 2004-000723-15; NCT00836654), cancer patients (*n* = 258) with recurrent symptomatic malignant ascites resistant to conventional chemotherapy were randomized to paracentesis plus catumaxomab (catumaxomab) or paracentesis alone (control) and stratified by cancer type (129 ovarian and 129 nonovarian). Catumaxomab was administered as an i.p. infusion on Days 0, 3, 7 and 10 at doses of 10, 20, 50 and 150 μg, respectively. The primary efficacy endpoint was puncture-free survival. Secondary efficacy parameters included time to next paracentesis, ascites signs and symptoms and overall survival (OS). Puncture-free survival was significantly longer in the catumaxomab group (median 46 days) than the control group (median 11 days) (hazard ratio = 0.254: *p* < 0.0001) as was median time to next paracentesis (77 *versus* 13 days; *p* < 0.0001). In addition, catumaxomab patients had fewer signs and symptoms of ascites than control patients. OS showed a positive trend for the catumaxomab group and, in a prospectively planned analysis, was significantly prolonged in patients with gastric cancer (*n* = 66; 71 *versus* 44 days; *p* = 0.0313). Although adverse events associated with catumaxomab were frequent, they were manageable, generally reversible and mainly related to its immunologic mode of action. Catumaxomab showed a clear clinical benefit in patients with malignant ascites secondary to epithelial cancers, especially gastric cancer, with an acceptable safety profile.

Malignant ascites results from the accumulation of fluid within the peritoneal cavity caused by the intraperitoneal (i.p.) spread of tumor cells^1^–^3^ and can be caused by a number of different cancers including ovarian, gastric, endometrial, breast, colon and pancreatic.^3^ The mechanisms leading to malignant ascites are independent of the primary tumor. While infiltrating the peritoneum, tumor cells interfere with the regulation of fluid flow in the peritoneal cavity. Because of an imbalance between efflux and influx, several liters of fluid can accumulate.^4^ Ascites results in poor quality of life because of burdensome symptoms such as abdominal swelling, a permanent feeling of fullness, pain, nausea, dyspnea, insomnia and fatigue.^1^,^5^–^7^ Malignant ascites is a manifestation of advanced disease and is associated with a poor prognosis.^3^,^8^,^9^ Although chemotherapy is the usual first-line treatment for patients presenting with ascites,^9^ there is a lack of evidence for its efficacy in patients with recurrent ascites from randomized trials. Intraperitoneal administration of radioisotopes or chemotherapy and peritoneovenous shunting procedures have also been used, but the data are limited.^10^,^11^ Paracentesis is the most common treatment for the relief of symptoms of recurrent ascites.^10^,^11^ However, repeated paracentesis leads to frequent hospital admission and is associated with several problems, including pain because of the procedure, protein loss causing hypovolemia and a risk of infection, peritonitis and bowel perforation. Furthermore, because paracentesis only provides short-term symptom relief, it has to be repeated frequently, often once a week.

Catumaxomab is a trifunctional monoclonal antibody with two different antigen-binding sites and a functional Fc domain.^12^ The two specific antigen-binding sites bind to epithelial tumor cells *via* the epithelial cell-adhesion molecule (EpCAM) and to T-cells *via* CD3. Furthermore, catumaxomab activates Fcγ-receptor I-, IIa- and III-positive accessory cells *via* its functional Fc domain.^13^ The simultaneous recruitment and activation of different immune effector cells at the tumor site leads to improved tumor-cell elimination by different immunologic killing mechanisms.^14^ EpCAM is expressed in the majority of epithelial cancers, making it an attractive target for antibody therapy.^15^ Tumor cells in malignant effusions have been shown to express EpCAM in 70–100% of those cases that commonly cause malignant ascites, *e.g*., breast, ovarian, gastric and colorectal cancer.^16^–^18^ EpCAM is also expressed on cells of normal epithelial tissues.^15^ However, normal EpCAM-positive tissue is assumed to be inaccessible to intact antibodies *in vivo* because of its protection by the basal lamina.^19^ In contrast, EpCAM in solid tumors is expected to be accessible for binding with intact antibodies after passage through the leaky tumor mosaic vessels or in body fluids such as ascites or pleural effusions. Furthermore, the peritoneal cavity is lined by mesothelial cells that do not express EpCAM.^20^ Therefore, the i.p. administration of catumaxomab offers the advantage of a targeted, locoregional immunotherapy against EpCAM+ tumor cells in the peritoneal cavity, which are the main cause of malignant ascites.

In previous studies, catumaxomab has demonstrated efficacy in patients with malignant ascites with an acceptable safety profile.^21^,^22^ This study is the first prospective, randomized trial designed to compare the i.p. infusion of catumaxomab plus paracentesis (C + P) with paracentesis alone to assess the efficacy and safety of catumaxomab in the treatment of malignant ascites due to epithelial cancers.

## Material and Methods

### Study design

**Figure 1:**
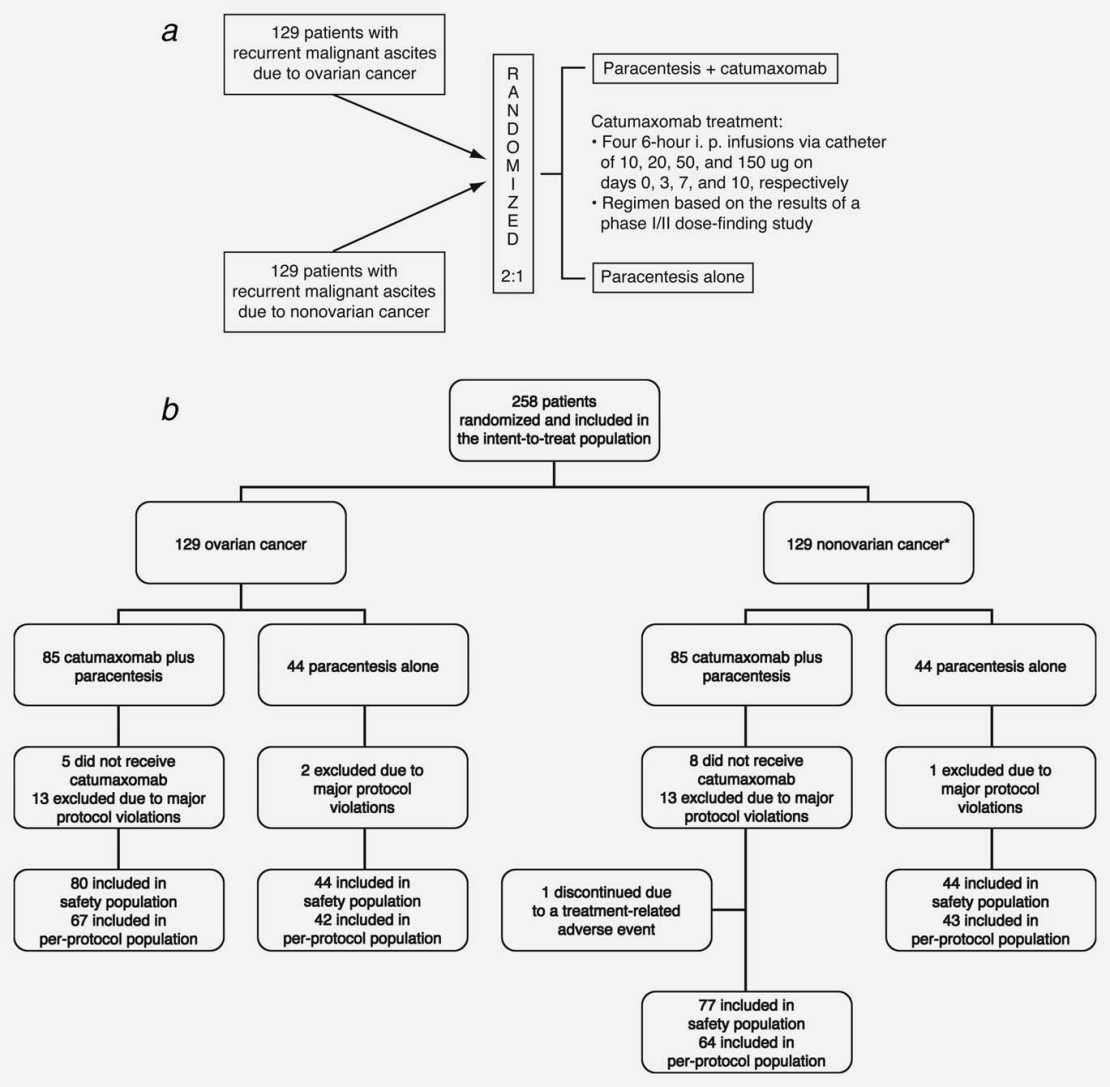
(*a*) Study design. (*b*) CONSORT flow diagram (only results for the intent-to-treat and safety populations are included in this article). *The main cancer types in the nonovarian stratum were gastric (*n* = 66, 51%), breast (*n* = 13, 10%), pancreas (*n* = 9, 7%), colon (*n* = 8, 6%) and endometrial (*n* = 6, 5%).

This was a two-arm, randomized, open-label, phase II/III study in patients with symptomatic malignant ascites secondary to epithelial cancers requiring symptomatic therapeutic paracentesis (Fig. 1). The study (EudraCT number: 2004-000723-15; ClinicalTrials.gov identifier: NCT00836654) was approved by an independent ethics committee at each study center, and all patients gave written informed consent before participation. The study was conducted in compliance with Good Clinical Practice guidelines and the Declaration of Helsinki.

### Treatment

After draining the ascites fluid, 500 mL of 0.9% sodium chloride solution was administered by i.p. infusion before each catumaxomab dose to support intra-abdominal distribution of the antibody. Based on the dose, catumaxomab was prediluted in an appropriate volume of 0.9% sodium chloride solution placed in a 50-mL perfusor syringe. Catumaxomab was administered as four 6-hr constant-rate i.p. infusions at doses of 10, 20, 50 and 150 μg on Days 0, 3, 7 and 10, respectively, *via* an i.p. catheter in parallel with an infusion of 250 mL of 0.9% sodium chloride solution. All catumaxomab i.p. infusions were performed in an inpatient setting. The dosing and administration regimen was based on the results of a phase I/II study.^22^ The catheter remained in the peritoneal cavity for all four infusions and was removed 1 day after the last infusion. Before each catumaxomab infusion and 1 day after the last infusion, the remaining fluid was drained from the peritoneal cavity *via* the catheter. The control group was treated with one therapeutic paracentesis only (Day 0).

In both groups, repuncture was performed if patients required relief of ascites symptoms. Investigators had a clear algorithm to determine when a therapeutic paracentesis should be performed. (*i*) Ascites volume >1 L based on a computed tomography (CT) scan was estimated by the local radiologist. This was retrospectively confirmed by two independent blinded readers and was supported by objective measurements of collected ascites volume and changes in body weight. The collected ascites volume was recorded and plotted against time to determine the *in vivo* accumulation rate. (*ii*) Symptomatic ascites based on signs (physical examination) and symptoms (ascites-specific patient questionnaire) was assessed by the investigator.

Patients were assessed with up to five follow-up visits at 8 days and 1, 3, 5 and 7 months (end of study) after the last infusion (catumaxomab group) or therapeutic paracentesis (Day 0, control group). In the control group, the end of the study was reached when the patient required the next paracentesis or died, whichever occurred first. For the analysis of the primary endpoint, any patients lost to follow-up were censored at the time of their last visit. After reaching the primary endpoint, all patients were further assessed every 2 months until death or 6 months after the last patient was randomized, whichever was later, for the evaluation of overall survival (OS). Patients in the control group who fulfilled the eligibility criteria and had two therapeutic punctures after Day 0 were permitted to receive catumaxomab in a subsequent, single-arm, crossover period (data not shown).

### Endpoints

The primary efficacy endpoint was puncture-free survival. This composite endpoint was defined as the time to first need for therapeutic puncture or death after treatment, whichever occurred first. Secondary efficacy parameters included time to next paracentesis, ascites signs and symptoms and OS. Ascites symptoms (anorexia, nausea, early satiety, vomiting, abdominal pain, abdominal swelling, dyspnea, fatigue, swollen ankles and heartburn) were assessed subjectively using a patient questionnaire with a four-point Likert scale (none, mild, moderate and severe).^23^ Ascites signs (abdominal distension dull to percussion, shifting dullness, fluid thrill and bulging flanks) were assessed objectively after abdominal examination by the investigator. The investigator also determined whether or not the ascites was symptomatic based on an assessment of the results of the patient questionnaire and the abdominal examination. For the final assessment of OS, the results of the poststudy period (*i.e*., the period after the primary endpoint was reached) were included.

Safety was assessed by adverse event reporting throughout the study. Adverse events were coded according to the Medical Dictionary for Regulatory Activities version 8.0. Adverse events were graded according to the National Cancer Institute Common Toxicity Criteria (version 2.0, April 30, 1999).

### Patients

Adult patients aged ≥18 years with histologically confirmed epithelial cancer and EpCAM+ tumor cells in the ascites fluid were eligible for inclusion in this study. Other inclusion criteria were as follows: Karnofsky performance status ≥60; life expectancy >8 weeks; estimated ascites volume >1 L by CT scan; at least one symptomatic paracentesis within 5 weeks as well as an objectively verified, clinical need for a second paracentesis and refractory or resistant to chemotherapy or standard chemotherapy was no longer feasible. Patients were excluded if they had: exposure to cancer chemotherapy or radiotherapy within the previous 28 days; previous treatment with murine monoclonal antibodies; enteral feeding at study entry or ileus within the previous 30 days and >70% tumor infiltration of the liver, or portal vein obstruction, or thrombosis. Patients were randomized 2:1 to paracentesis plus catumaxomab or paracentesis alone (control) and stratified by cancer type (ovarian and nonovarian).

### Determination of tumor-cell load

Tumor-cell load (number of EpCAM+ tumor cells/10^6^ ascites cells) in ascites fluid was analyzed for both groups at screening and at puncture visit. In addition, measurements were performed in the catumaxomab group during treatment before the second infusion and 1 day after the last infusion. Tumor-cell load was determined *via* quantification of EpCAM+ tumor cells in ascites fluid/peritoneal lavage and was performed using immunocytochemistry as described elsewhere.^21^ Briefly, ascites cells were harvested by centrifugation or ficoll density centrifugation. A constant number of cells was centrifuged on cytospin slides, and tumor cells were labeled at screening (before therapy) with the anti-EpCAM antibody HO-3 (the parental antibody of catumaxomab; TRION Pharma, Munich, Germany), ensuring that the patients' EpCAM+ tumor cells could be recognized by catumaxomab. To prevent inhibition of antibody staining by catumaxomab residuals in ascites samples during and after therapy, these samples were stained with the anti-EpCAM antibody VU1D9 (TRION Research, Martinsried, Germany), which recognizes a different EpCAM epitope to HO-3/catumaxomab. HO-3 and VU1D9 were both directly labeled with a Texas Red fluorescence dye. All cytospins were analyzed by a computerized image analysis system (MDS™; Applied Imaging International, Newcastle upon Tyne, UK) to count the cells labeled with Texas Red. Leukocytes were stained with an anti-CD45 antibody (Caltag Laboratories, Hamburg, Germany) and its corresponding secondary antibody IgG1 Alexa Fluor 488 (Molecular Probes, Invitrogen, Karlsruhe, Germany).

### Detection of antidrug antibodies

As catumaxomab is a nonhumanized mouse/rat antibody, the development of human anti-mouse antibodies (HAMAs) against catumaxomab was investigated. HAMAs were analyzed in serum samples at the following timepoints: at screening, before the third infusion, before the fourth infusion, 8 days and 1 month after the last infusion and at puncture visit. A commercially available *in vitro* diagnostic test, HAMA-ELISA Medac (Medac, Hamburg, Germany), a simple and rapid one-step enzyme immunoassay for the quantitative determination of HAMAs in serum, was used. The test was performed according to the manufacturer's instructions and calibrated against goat anti-mouse IgG antibodies, with a measuring range of 40–2,000 ng/mL. Briefly, microtiter plates were coated with mouse IgG and then clinical-trial samples and peroxidase-labeled mouse IgG (conjugate) were added. For detection of bound HAMAs, a tetramethylbenzidine substrate was added. The reaction was stopped by the addition of sulfuric acid. The absorption of the colored product was measured photometrically at 450 nm (reference wavelength: 620–650 nm). An internal test to detect HAMAs and human anti-rat antibodies showed a significant correlation with the results of the Medac test.

### Statistics

The planned sample size of 216 randomized patients was based on the following considerations: the primary efficacy variable (puncture-free survival), the duration of patient observation (7 months [30 weeks]), the median time to end of puncture-free survival (twice the time in the catumaxomab group than in the control group: the assumed median puncture-free survival time was 5 weeks in the control group *versus* 10 weeks in the catumaxomab group), 10% of patients lost to follow-up, alpha level 0.05, two-sided, no adjustment for the two tests (ovarian and nonovarian subgroups) and a randomization ratio of 2:1 (catumaxomab *versus* control group).

All statistical tests were two-sided, and the level of significance was 5%. The evaluation of puncture-free survival in the intent-to-treat (ITT) population (all randomized patients) was the primary efficacy analysis. The log-rank test and the hazard ratio (HR) were used to compare puncture-free survival between the two treatment groups. The variables to define the need for paracentesis were analyzed by chi-square test, kappa coefficients, McNemar's test and analysis of covariance (ANCOVA), as appropriate. Secondary variables were analyzed with Kaplan-Meier estimates, log-rank test, HR, chi-square test, Cox regression analysis, Cochran-Mantel-Haenszel test, ANCOVA or Wilcoxon test, as appropriate. Statistical testing for secondary variables was descriptive only. The safety population consisted of all patients who received at least one dose of catumaxomab or were randomized to the control group. Data were analyzed separately for patients with ovarian and nonovarian cancer with an additional analysis of the pooled population and a subgroup population of the nonovarian cancer patients with gastric cancer (the largest patient subgroup in the nonovarian cancer stratum).

## Results

From September 2004 to August 2006, 258 patients were randomized at 75 centers in 13 countries (ITT population): 85 to paracentesis plus catumaxomab (catumaxomab) and 44 to paracentesis alone (control) in both disease strata (129 ovarian and 129 nonovarian cancer) (Fig. 1). The main cancer types in the nonovarian stratum were gastric (*n* = 66, 51%), breast (*n* = 13, 10%), pancreas (*n* = 9, 7%), colon (*n* = 8, 6%) and endometrial (*n* = 6, 5%). Thirteen patients in the catumaxomab group (five ovarian and eight nonovarian cancer) were not treated and were withdrawn from the study, but were included in the ITT population. The reasons for not receiving catumaxomab treatment were withdrawal of consent (*n* = 5), death before treatment could be started (*n* = 4) and other (*n* = 4), which included a serious adverse event, ileus, failure to meet inclusion criteria and problems with ascites drainage. All 88 patients in the control group received a therapeutic paracentesis at Day 0. Three patients in the control group (all nonovarian cancer) were lost to follow-up. In total, 245 patients (157 catumaxomab and 88 control) were included in the safety population. More than 80% of these patients (86% [*n* = 69] ovarian and 81% [*n* = 62] nonovarian cancer) received all four planned infusions. Reasons for patients receiving less than four infusions included adverse events (*n* = 20), withdrawal of patient consent (*n* = 3) and other/not known (*n* = 3). Sixty-one percent (58% catumaxomab; 64% control) of patients had distant metastases and 22% (21% catumaxomab; 23% control) of these patients had liver metastases. There were no relevant differences in baseline characteristics between the treatment groups in either strata (Table 1).

**Table 1:** Demographic and clinical characteristics at baseline (intent-to-treat population)

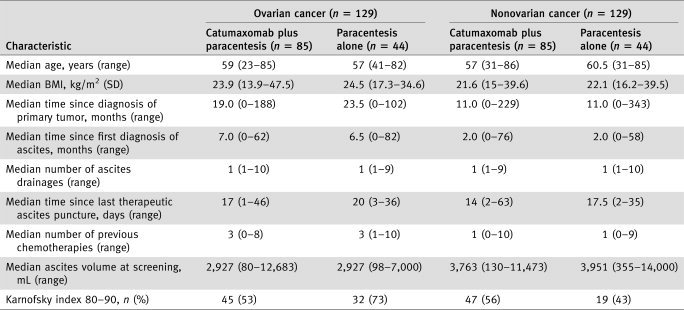

**Figure 2:**
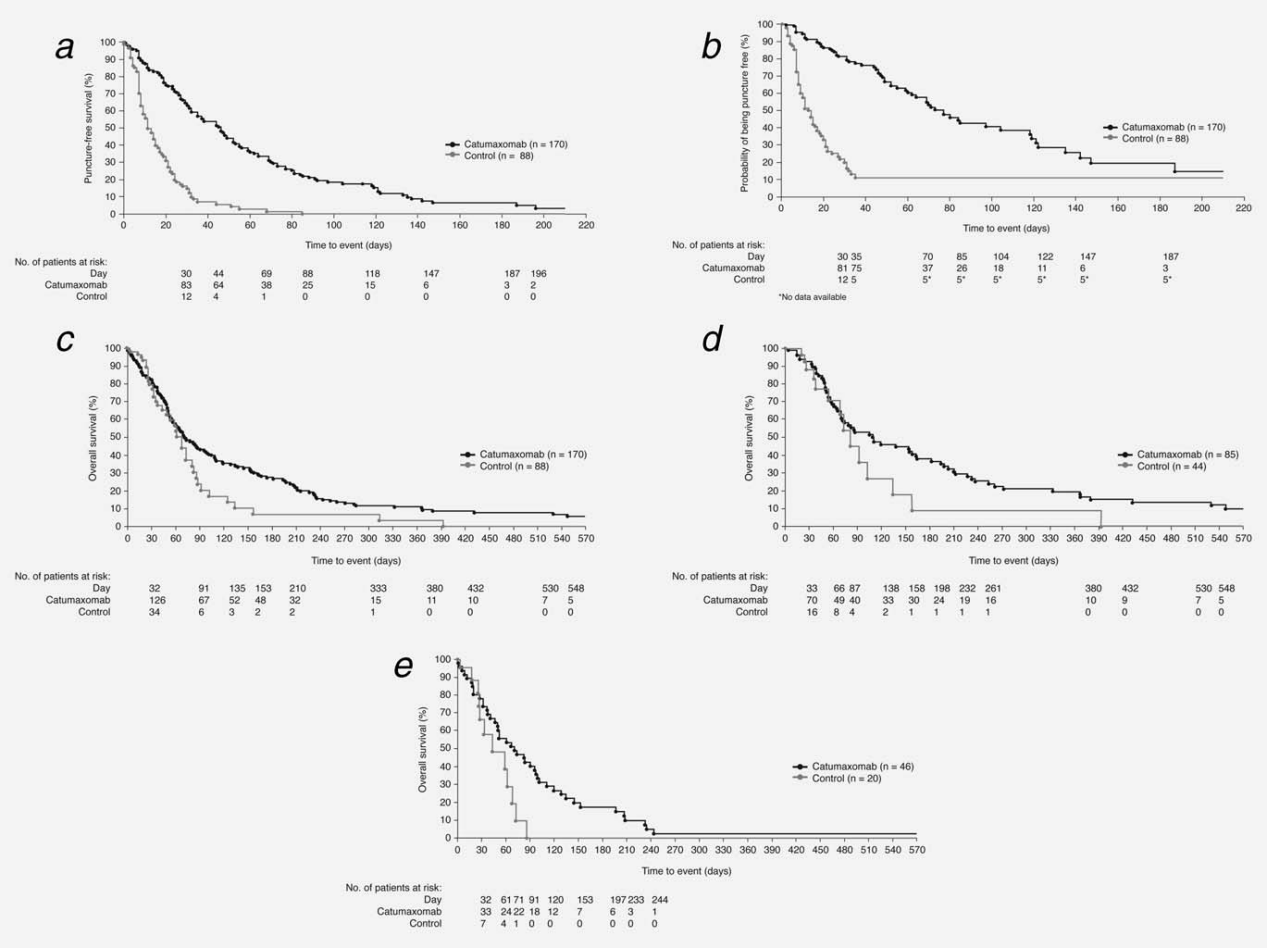
Kaplan-Meier estimates of puncture-free survival, time to next paracentesis and overall survival (intent-to-treat population). (*a*) Puncture-free survival in the pooled population; (*b*) time to next paracentesis in the pooled population; (*c*) overall survival in the pooled population; (*d*) overall survival in patients with ovarian cancer and (*e*) overall survival in patients with gastric cancer.

Median puncture-free survival was longer in catumaxomab patients in both strata and therefore also in the pooled analysis (46 days) compared to the control (11 days) (Fig. 2*a*). In the ovarian and nonovarian cancer patients, median puncture-free survival was 52 and 37 days for paracentesis plus catumaxomab *versus* 11 and 14 days for paracentesis alone, respectively (Table 2). All differences were statistically significant (*p* < 0.0001). The HR for the pooled population was 0.254 (95% confidence interval [CI] 0.185–0.350, Table 2), corresponding to a risk reduction for puncture or death of 75%. For the determination of puncture-free survival, 66% of the events (133/201) were punctures and 34% (68/201) were deaths. Puncture-free survival was also determined separately for patients with and without distant metastases, in particular liver metastases, and an increase was seen with catumaxomab *versus* paracentesis alone: with metastases, 44 *versus* 13 days; without metastases, 48 *versus* 11 days; with liver metastases, 27 *versus* 9 days and without liver metastases, 49 *versus* 14 days (*p* < 0.0001 for all comparisons).

**Table 2:** Puncture-free survival, time to next paracentesis and overall survival

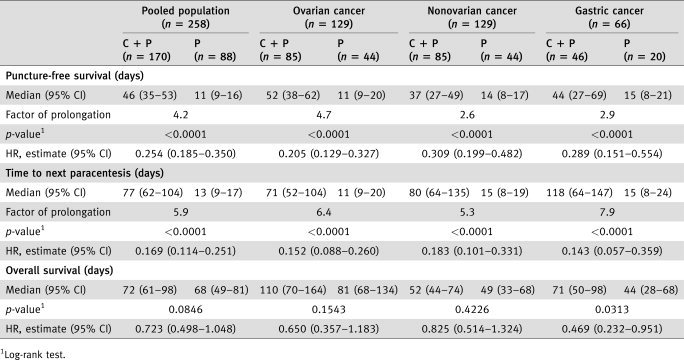

The median time to the next therapeutic puncture was also significantly longer in the catumaxomab group compared to the control: pooled population, 77 *versus* 13 days (HR 0.169; 95% CI 0.114–0.251) (Fig. 2*b*); ovarian cancer, 71 *versus* 11 days (HR 0.152; 95% CI 0.088–0.260) and nonovarian cancer, 80 *versus* 15 days (HR 0.183; 95% CI 0.101–0.331; *p* < 0.0001 for all comparisons) (Table 2). The largest difference in median time to next paracentesis was observed in gastric cancer patients, the largest subpopulation of nonovarian cancer patients: 118 *versus* 15 days (HR 0.143; 95% CI 0.057–0.359; *p* < 0.0001).

**Figure 3:**
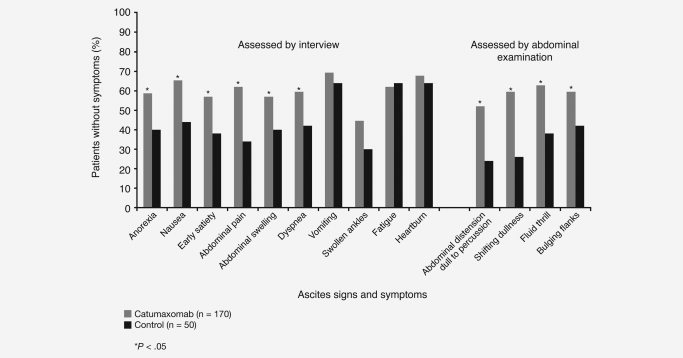
Patients without ascites signs and symptoms as assessed by interview 8 days after last infusion (catumaxomab) or 8 days after paracentesis (control group).

Measures implemented to objectify the timepoint of paracentesis showed the following results. CT scans were performed by the local radiologist for the majority of patients (catumaxomab: 67%; control: 83%) to estimate whether the ascites volume was >1 L at the puncture visit. The concordance between the initial CT estimation and the results of a blinded reader was >80% in all patient groups and at all timepoints. In >70% of patients, the ascites volume collected at the puncture visit was >2 L. In the catumaxomab and control groups, the median collected ascites volumes were 3,500 *versus* 3,251 mL, respectively, corresponding to body weight changes of −3.0 kg for catumaxomab patients and −2.8 kg for control patients. There was a statistically significant difference in the median calculated daily ascites volume collected in the catumaxomab *versus* the control group: 82 mL/day *versus* 271 mL/day in the ovarian cancer stratum (*p* = 0.0006) and 55 mL/day *versus* 414 mL/day in the nonovarian cancer stratum (*p* < 0.0001). The median percentage of patients with reported symptomatic ascites at puncture visit was 92% in the catumaxomab group *versus* 95% in the control group. In both groups, ascites signs and symptoms at screening and puncture visit were comparable. There were fewer ascites signs and symptoms (assessed 8 days after the last catumaxomab infusion or after Day 0 in the control group) in catumaxomab patients compared to the control group (Fig. 3). In six of ten symptom categories (anorexia, nausea, early satiety, abdominal pain, abdominal swelling and dyspnea) and in all four sign categories (abdominal distension dull to percussion, shifting dullness, fluid thrill and bulging flanks), these differences were statistically significant (*p* < 0.05).

The median OS (secondary endpoint) was 72 days for paracentesis plus catumaxomab compared to 68 days for paracentesis alone (*p* = 0.0846) in the pooled analysis (Fig. 2*c*, Table 2). In the ovarian (Fig. 2*d*) and nonovarian cancer strata, the median OS was 110 and 52 days for paracentesis plus catumaxomab and 81 and 49 days for paracentesis alone, respectively (ovarian cancer: *p* = 0.1543; nonovarian cancer: *p* = 0.4226) (Table 2). The difference in OS was statistically significant in patients with gastric cancer (median 71 *versus* 44 days; *p* = 0.0313) (Fig. 2*e*, Table 2). The HR for the pooled population was 0.723 (95% CI 0.498–1.048), corresponding to a risk reduction for death of 27.7%.

**Figure 4:**
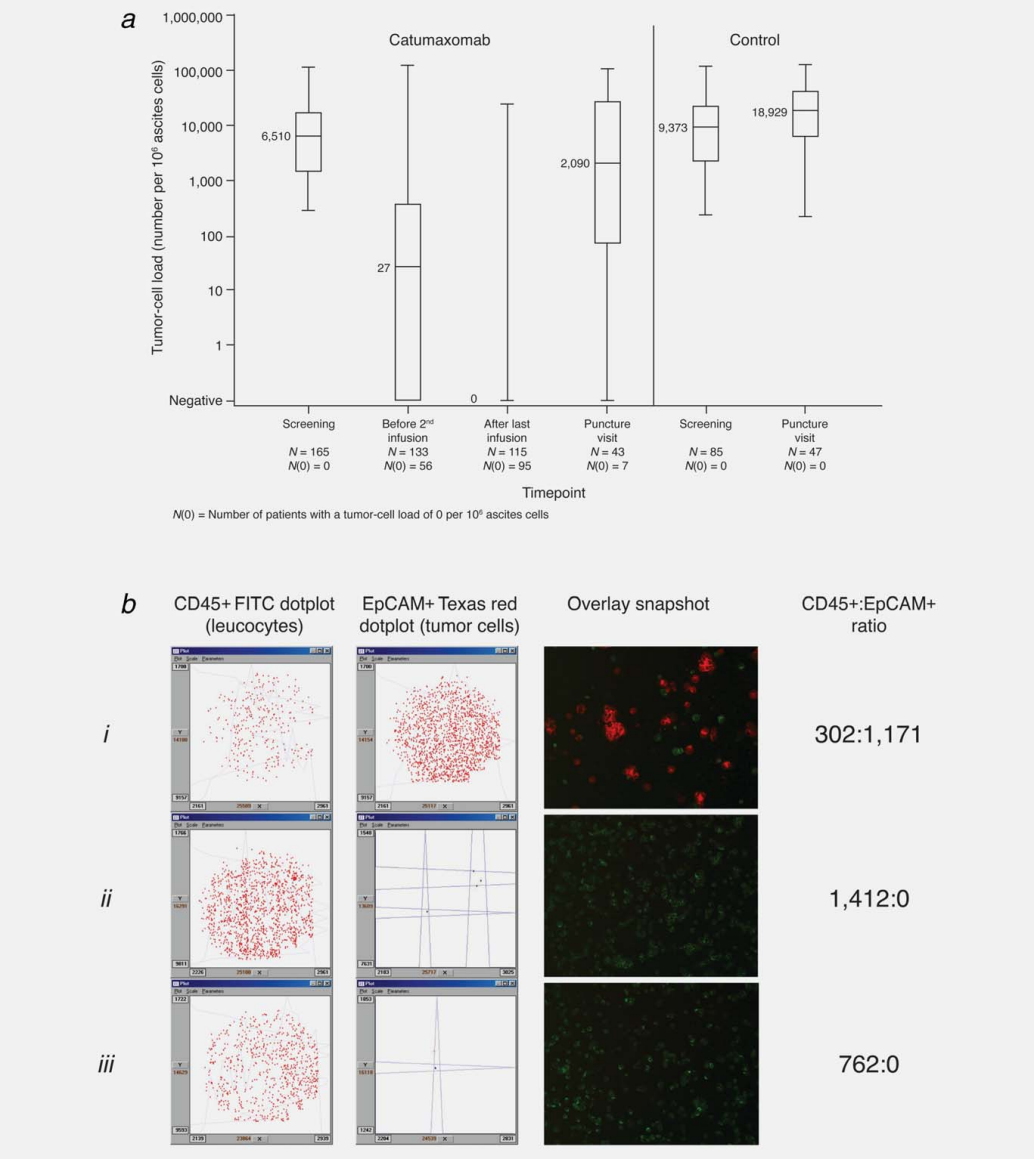
(*a*) Tumor-cell load in the ascites fluid before, during and after catumaxomab treatment. Number of EpCAM+ tumor cells per 10^6^ total cells evaluated by immunocytochemistry. The median tumor-cell count at the repuncture visit was statistically significantly lower (*p* = 0.0012) in the catumaxomab group. (*b*) Fluorescent double staining of ascites fluid cells: evaluation of CD45+ leukocyte:EpCAM+ tumor cell ratio during catumaxomab therapy. (*i*) Malignant ascites harvested at screening puncture (before catumaxomab treatment). (*ii*) At Day 3 after 10 μg catumaxomab i.p. (*iii*) At Day 11 after a total of 230 μg catumaxomab i.p. Dotplot analysis: every plot resembles a fluorescence-labeled cell that was detected and counted by the computerized image analysis system.

EpCAM+ tumor cells in the ascites fluid were significantly reduced after catumaxomab treatment to a median of zero after the last infusion (pooled population, Fig. 4*a*): 95/115 patients had a tumor-cell count of zero after the last infusion. In contrast, the number of CD45+ leukocytes was increased in ascites/lavage samples during and at the end of catumaxomab treatment (Fig. 4*b*). The median tumor-cell count at the repuncture visit was statistically significantly lower (*p* = 0.0012) in the catumaxomab group (2,090 tumor cells/10^6^ analyzed cells; median 77 days after treatment) than in the control group (18,929 tumor cells/10^6^; median 13 days after paracentesis). Furthermore, the median volume of ascites fluid produced was also reduced after catumaxomab treatment. The median ascites volume drained after treatment with catumaxomab minus the volume of the first paracentesis and the volume infused (∼800 mL per infusion) was 645 mL. Before the second and third infusions, the median drained volume was greater than the volume infused, whereas after the fourth infusion, the median volume drained was similar to the volume infused (750 and 782 mL, respectively).

The safety population included 157 catumaxomab patients and 88 control patients. As catumaxomab was effective in prolonging puncture-free survival, the observation period for adverse events was distinctly longer in the catumaxomab than in the control group. Thus, comparison of adverse-event frequencies between the treatment groups is misleading. The pattern of adverse events in the ovarian and nonovarian cancer patients was similar, irrespective of the adverse events related to the primary tumor. More than 98% of catumaxomab patients had at least one adverse event and 85% had adverse events considered related to study treatment (Table 3). There was no distinctive pattern of adverse events corresponding to specific infusions. Ninety-one patients (58%) experienced serious adverse events, of whom 23 (15%) had serious adverse events considered to be treatment related.

**Table 3:** Adverse events considered related to catumaxomab occurring in ≥5% of patients (*N* = 157)

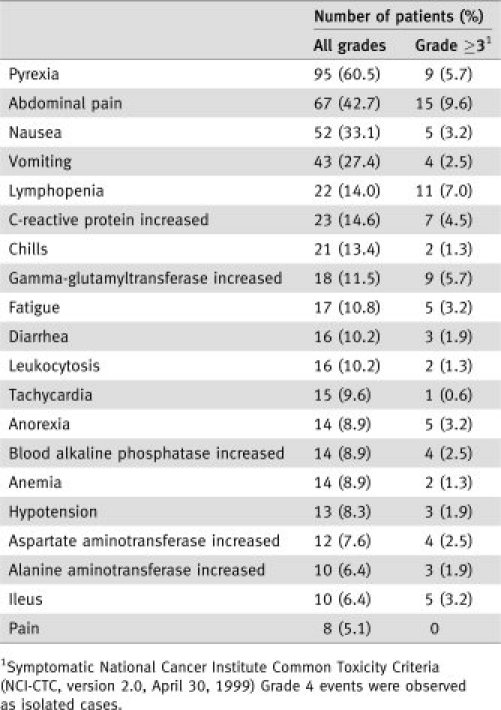

The most commonly reported catumaxomab-related adverse events in both the ovarian and nonovarian groups were cytokine release–related symptoms (pyrexia, nausea and vomiting) and abdominal pain. These events were generally mild to moderate in intensity and reversible. There were no cases of catheter-related infections. Ten patients experienced ileus (Grade 1: *n* = 1; Grade 2: *n* = 4; Grade 3: *n* = 2 and Grade 4: *n* = 3), and three patients experienced subileus events (Grade 2: *n* = 2 and Grade 3: *n* = 1). Of these patients, seven cases of ileus (Grade 2: *n* = 2; Grade 3: *n* = 2 and Grade 4: *n* = 3) and two cases of subileus (both Grade 2) were reported as serious adverse events. One patient had a Grade 3 gastric hemorrhage also reported as a serious adverse event.

There were 61 deaths (26 ovarian and 45 nonovarian cancer) during the study, none of which were considered related to catumaxomab. Laboratory parameters showed similar trends in ovarian and nonovarian cancer patients. Abnormal liver parameters (transient increases in alanine aminotransferase, aspartate aminotransferase, alkaline phosphatase and gamma-glutamyltransferase) and white blood cell disorders (lymphopenia, leukocytosis and anemia) were regularly observed. However, these laboratory abnormalities were rarely considered clinically relevant and were generally reversible.

Before treatment with catumaxomab, plasma samples from the vast majority (>95%) of patients were HAMA negative. Before the fourth infusion, most patients (91% [59/65] ovarian cancer and 98% [58/59] nonovarian cancer) were still HAMA negative. Eight days after the last catumaxomab infusion, 82% (49/60) of ovarian cancer patients and 69% (36/52) of nonovarian cancer patients were HAMA positive, whereas 1 month after the fourth infusion, 95% (36/38) of ovarian cancer patients and 94% (30/32) of nonovarian cancer patients had become HAMA positive. In patients with a positive HAMA test before the fourth infusion (*n* = 7), there were no obvious changes in the pattern of adverse events reported after the last infusion.

## Discussion

There is a great need for an effective treatment of malignant ascites. Although some novel therapeutic approaches have been tried over the last 2 decades for the treatment of malignant ascites, none have proved to be effective. These approaches include the i.p. administration of interferon-α,^24^,^25^ tumor necrosis factor (TNF)-α,^26^,^27^ matrix metalloproteinase inhibitors,^28^ nonpathogenic infectious agents^29^ and vascular endothelial growth factor inhibitors.^30^,^31^ However, most of the studies were case reports, pilot studies or phase I trials. The only randomized study, which compared i.p. TNF-α plus paracentesis with paracentesis alone, showed no improvement with the addition of TNF-α.^26^ To date, there have been no randomized clinical trials demonstrating the efficacy of chemotherapy, and there is no accepted standard treatment for symptom relief other than repeated paracentesis. This study is the first prospective, randomized clinical trial in malignant ascites to demonstrate a beneficial effect of treatment. The results demonstrate that paracentesis plus catumaxomab had superior efficacy compared to paracentesis alone for the treatment of malignant ascites in patients with epithelial cancer. Immunotherapy with catumaxomab or bispecific antibodies has previously been shown to be effective in preventing and/or diminishing the accumulation of ascites and eliminating tumor cells with an acceptable safety profile in patients with advanced ovarian cancer.^21^,^22^,^32^

The primary endpoint of our study was puncture-free survival. This composite endpoint was chosen because patients with symptomatic malignant ascites have a very short life expectancy, usually only weeks to months. The statistically significant and clinically relevant prolongation of puncture-free survival for paracentesis plus catumaxomab compared to paracentesis alone in both ovarian and nonovarian cancer patients indicates that catumaxomab treatment is beneficial for a number of different types of epithelial cancer. Although puncture-free survival provides the most reasonable estimate of the treatment effect in this patient population with advanced disease, the secondary endpoint of time to next paracentesis (a component of the primary endpoint) mainly reflects the clinical relevance for the individual patient.

The time to next paracentesis was also significantly prolonged for paracentesis plus catumaxomab *versus* paracentesis alone in both strata. The objective measurements used to verify the puncture timepoint, *e.g*., ascites volume, confirmed the need for therapeutic puncture. A comparison of the median time to the next therapeutic puncture in the control group (13 days) with that in the catumaxomab group (77 days) indicates that catumaxomab treatment avoided the need for approximately five punctures. This is clinically relevant, considering the continuous protein loss and the potential risk for infection or bowel perforation with each puncture. In addition to benefiting from fewer punctures, patients benefit from a fivefold longer time without burdensome and persistent ascites symptoms, such as abdominal pain, abdominal pressure, dyspnea, nausea, vomiting and early satiety. By controlling ascites symptoms, catumaxomab helps to improve patients' quality of life and enables patients with end-stage disease to lead a more normal daily life.

Although treatment benefits with regard to OS are unlikely in such advanced disease and the study was not powered or designed to detect a difference in OS, the Kaplan-Meier curves indicate that catumaxomab prolonged OS in ∼50% of patients. In particular, a subgroup analysis of gastric cancer patients, the largest subpopulation in the nonovarian stratum, demonstrated a statistically significant prolongation of OS. One reason for this observation could be that nonovarian cancer patients, including those with gastric cancer, had fewer previous chemotherapies (median of one) compared to ovarian cancer patients who received a median of three previous chemotherapies. This higher degree of treatment experience may have resulted in a less functional immune system, with a consequent impairment of catumaxomab's mode of action. Catumaxomab treatment also significantly reduced the number of tumor cells, which are the main cause of malignant ascites,^4^ during and after treatment. Interestingly, the number of tumor cells remained substantially lower until the time of repuncture compared to the control group, demonstrating the long-lasting immunologic antitumor effect induced by catumaxomab. Taken together, all the efficacy parameters indicate consistent results and show a clear clinical benefit for catumaxomab in patients with recurrent malignant ascites for whom no other standard therapy apart from paracentesis has been established, irrespective of the primary epithelial cancer.

Catumaxomab had acceptable tolerability without unexpected severe or long-lasting adverse events. More than 80% of patients received the complete dose, which is rarely seen with other i.p. therapies.^33^ The safety results of this study confirm the specific pattern of the commonly observed catumaxomab adverse events that are mainly related to its immunologic mode of action.^22^ Symptoms (*e.g*., pyrexia, vomiting and nausea) associated with the release of proinflammatory, modulatory and cytotoxic cytokines were very common and are a well-known consequence of antibody therapy in general.^34^,^35^ The ascending dose schedule of four infusions used in this study was selected to improve the tolerability of catumaxomab, as early studies indicated that patients did not tolerate high starting doses because of symptoms related to cytokine release.^22^ However, cytokine-release-related symptoms may be positively correlated with efficacy and may serve as a predictive factor for the efficacy of catumaxomab.^36^ The frequent occurrence of abdominal pain with catumaxomab is considered to be partly a symptom of peritoneal irritation following i.p. administration.

Although the adverse events associated with catumaxomab were frequent, they were manageable and generally reversible. The alterations in laboratory parameters were rarely considered clinically relevant. Transiently increased liver parameters were typically observed, which can be partially explained by catumaxomab binding to EpCAM+ bile duct cells and the release of cytokines.^37^ The transient decrease in the peripheral lymphocyte count might be the result of migration of lymphocytes into the tissue and possibly to the tumor, mediated by upregulation of adhesion molecules on white blood cells after antibody-mediated activation.^38^

The development of HAMAs in response to the administration of murine antibodies has been reported in the literature.^32^,^39^ Therefore, as expected, most of the patients in this study developed antibodies against the nonhumanized mouse/rat antibody catumaxomab. Interestingly, elevated HAMA levels after treatment of ovarian cancer patients with the bispecific F(ab)2 OC/TR antibody were associated with longer median survival.^40^

In conclusion, treatment with four i.p. doses of C + P demonstrated clinically relevant benefits in patients with recurrent malignant ascites due to carcinomas of different origin. Positive trends in OS together with its demonstrated efficacy against tumor cells in the peritoneal cavity support the antitumor activity of catumaxomab and suggest that it could be even more effective if used at an earlier stage in the treatment of epithelial cancers. Catumaxomab showed a typical pattern of adverse events that are mainly related to its immunologic mode of action. However, these are both, manageable and generally reversible. The i.p. administration of catumaxomab can be regarded as a promising new therapy for malignant ascites.

## Abbreviations:

ANCOVA: analysis of covariance
C + P: catumaxomab plus paracentesis
CI: confidence interval
CT: computed tomography
EpCAM: epithelial cell-adhesion molecule
HAMA: human anti-mouse antibody
HR: hazard ratio
i.p.: intraperitoneal
ITT: intent-to-treat
OS: overall survival
P: paracentesis alone
TNF: tumor necrosis factor
